# Effect of Heat Treatment on the Physic-Mechanical Characteristics of *Eucalyptus* *urophylla* S.T. Blake

**DOI:** 10.3390/ma14216643

**Published:** 2021-11-04

**Authors:** Lin Yang, Honghui Jin

**Affiliations:** 1 College of Furnishings and Industrial Design, Nanjing Forestry University, Nanjing 210037, China; JHongHui@njfu.edu.cn; 2 Co-Innovation Center of Efficient Processing and Utilization of Forest Resources, Nanjing Forestry University, Nanjing 210037, China

**Keywords:** eucalyptus, heat treatment, dimensional stability, physic-mechanical property, wood color

## Abstract

Eucalyptus plantations wood have great potential application in high quality solid wood product. In order to improve the overall characteristics, heat treatments (HT) were carried out using *Eucalyptus urophylla* S.T. Blake wood at 150, 170 and 190 °C, for 2 and 4 h, separately. The effects of HT on physical and mechanical properties, wood color, dimensional stability and chemical change were investigated. The results indicate that: Mass loss (*ML*) of wood at a moderate temperature of 150 °C was small, but increased remarkably when temperature exceeds 170 °C. A maximum *ML* of 5.83% was observed at 190 °C/4 h; the velocity of water vapor adsorption and equilibrium moisture content (*EMC*) of HT wood decreased significantly, and varied considerably with treating severity; absolute dry density of HT wood decreased, presenting a similar tendency with *ML*, but the reduction was greater than *ML*; HT reduced the tangential and radial swelling ratio and swelling coefficients of wood, and improved the dimensional stability by 71.88% at 190 °C; modulus of rupture (MOR) and modulus of elasticity (MOE) of HT wood varied significantly in severer conditions, but there were no obvious changes in a moderate conditions at 150 °C; there was a slight color change at 150 °C, but wood color became more dark and uniform with treating severity; HT decreased the relative content of hydroxyl groups in wood components, improving wood dimensional stability. Color change of wood may be caused by variations of chromophoric groups and its own structure of lignin due to HT. Moderate temperature HT at 150 °C improved dimensional stability and color uniform of wood, but without reducing mechanical stress. This is a practical HT condition for *Eucalyptus urophylla* S.T. Blake.

## 1. Introduction

Eucalyptus plantations are planted widely in south China owing to their advanced growth characteristics, such as good response to fertilization [1], pests’ resistance [2], strong adaptation to environment [3], and are the predominant species of industrial fast-wood plantation in the country. Some eucalyptus plantations are fast growing which results in poor physical-mechanical performance and generally are used to make material in pulp and paper, wood-based panels, and packing box production. Meanwhile, some new technologies and researches have been also undertaken to improve further application for wood material [4,5,6]. However, several Eucalyptus woods are required by the timber industry because they have relatively good mechanical performance, excellent productivity, few knots, and good glued joints performance, which have been considered as the technological properties for high quality solid wood products, such as those produced in the furniture and civil construction industry [7,8,9]. Despite the advantages above, Eucalyptus wood has some undesirable properties such as drying problems, high swelling, low dimensional stability, and unattractive color patterns, which limit its further use for high value-added solid wood products.

Suitable drying technology can reduce drying stress and improve wood quality [10,11], but for Eucalyptus wood, further treatments are needed to obtain better quality. In order to overcome such disadvantages and increase wood properties, the use of technological modification treatments have been developed. Among these modifications, heat treatments (HT), as an environmentally friendly modification, have been commercially applied to wood industry [12]. The HT woods are applied for exterior and interior uses such as decks, fences, garden and kitchen furniture, parquet, etc. [13]. HT changes wood chemical compositions by degrading cell wall compounds and altering extractives leading to mass loss and changes of wood structure [14,15]. Generally, HT affects wood chemical composition improving its physical characteristics, hygroscopicity, dimensional stability, and durability [16,17,18,19,20]. In previous studies, effects on wood properties were investigated for many conifer and broadleaf trees, such as eucalyptus, poplar, oak, birch, spruce, and Scots pines with HT applied at various temperature and duration. These studies showed that, with increasing temperature and duration, shrinking and swelling reduces up to 55–90%, equilibrium moisture content (*EMC*) reduces by 50%, some mechanical properties decrease by 0–30%, wood color generally changes to brown or dark and presents various color stability, and biological durability increase [21,22,23,24].

Many heat treatments have been increasingly employed to Eucalypt plantation woods to improve their performance for high quality solid wood products [25,26]. The mechanical properties, color, and dimensional stability are important characteristics for Eucalypt plantation wood used as solid wood material. Many studies indicated that mechanical properties reduce after severe HT, with the exception of MOE. Santos’s test showed that MOE of *Eucalyptus*.*globulus* wood was higher than that of control specimens when wood HT at 180 °C [27]. Another study showed that there were no differences between the MOE groups for *Eucalyptus grandis* treated at temperature between 140 to 220 °C [28]. However, some other studies reported that MOE vary between tree species, temperature, and duration of treatment [29,30]. The color compatibility of components of wood after HT is a major criterion to evaluate the quality of furniture, door, and flooring [31]. Color of HT wood becomes darker and uniform throughout the thickness [14,32], which is benefit for value-added solid wood product. However, color changes depend on temperature, duration, and techniques [33]. Moreover, decreased dimensional stability and increased durability are some of the main findings for heat treated Eucalyptus woods [34,35,36]. Mechanical properties, color, and dimensional stability of wood are affected by the thermal degradation of wood composition, and severe treatments result in high loss of mechanical properties and significant color changes limiting the use of this material for high value-added solid wood products.

Eucalyptus plantations, a fast-growing tress, are not suitable for many applications. However, as one of the least expensive woods, there is a significant interest in heat treating it to enhance its dimensional stability, reduce its color change, and increase its biological durability, but without negatively impacting the strength properties. Thus, in this study, *Eucalyptus urophylla* S.T. Blake wood were heat treated at 150, 170, and 190 °C, for 2 and 4 h, respectively. Our objective was to systemically determine the effect of HT on some physical and mechanical properties, wood color, dimensional stability, and chemical change in *Eucalyptus urophylla* S.T.

## 2. Materials and Methods

### 2.1. Materials

The *Eucalyptus urophylla* S.T. Blake in this survey were obtained from Guangxi Provence, China, which were eight-year-old plantation-grown tress having an average annual ring width of 3.5 mm and a basic density of 0.513 g·cm^−3^. The trees were produced into 1000 mm (L) logs and air dried in the wood LAB of Nanjing Forestry University for 1 year prior to tests. Thereafter, the logs were sawn and planed into small lumbers with dimensions of 20 (R) × 20 (T) × 1000 (L) mm^3^. Then, the small lumbers were produced into specimens with two dimensions of 20 (R) × 20 (T) × 2 0(L) mm^3^ and 20 (R) × 20 (T) × 300 (L) mm^3^. The large specimens were used for mechanical test and color measurement. The initial mass and dimensions were measured after specimen preparation, which were used for determination of moisture content and density, etc. Defect free specimens were selected and the initial MCs of all specimens were about 11.5%. 

### 2.2. Heat Treatment

Prior to HT, all specimens were first dried at 60 °C for 8 h and then at 103 °C until constant mass were obtained, and then the mass, dimensions, and color of oven-dried specimens were measured. Thereafter, specimens were randomly divided into seven treatment groups in addition to one control group. Seven replicates in each treatment group were heat treated under atmospheric pressure in a lab-used chamber (XN-TH100, Jiangsu Xingnan Drying Equipment Co., ltd., NanJing, China) at 150, 170, and 190 °C, for 2 and 4 h, respectively. The HT specimens were cooled down to room temperature in a sealed Ziploc bag. Finally, the mass, dimensions and color of HT wood were measured again.

### 2.3. Mass Losss (ML)

The *ML*s of treated specimens were calculated from the mass changes according to Equation (1) using the small samples of 20 (R) × 20 (T) × 20 (L) mm^3^.

*ML* = (*M*_o_ − *M*_h_)/*M*_o_ × 100% (1)

where *M*_o_ (g) and *M*_h_ (g) are the oven-dried mass of specimens prior to and after HT, respectively.

### 2.4. Moisture Adsorption

These properties were determined according to GB/T-1931. The oven-dried specimens of treated and control wood were kept at 20 ± 2 °C and 65 ± 3% relatively humidity (RH) in a conditioning chamber until weight constancy was achieved. During the moisture adsorption process, mass, and dimensions in tangential and radial direction of all specimens were measured by an electronic balance (JA21002) (Shanghai Liangping Instrument and Meter Co., Ltd., Shanghai, China) and a digital caliper (CD-20CPX) (Mitutoyo, Kawzaki, Japan). The capacity of treated and control wood for moisture adsorption are described by equilibrium moisture content (*EMC*), which are calculated according to Equation (2).

*EMC* = (*M*_e_ − *M*_o_)/*M*_o_ × 100% (2)

where *M*_o_ (g) is the initial mass of oven-dried specimens, *M*_e_ (g) is the mass after equalization in the conditioning chamber.

### 2.5. Density

Density of wood depends on water in specimens. Therefore, density of wood at oven-dried conditions (*D*_o_) was calculated in this study to evaluate the influence of HT according to Equation (3).

*D*_o_ = *M*_o_/*V*_o_ × 100% (3)

where *M*_o_ (g) and *V*_o_ (cm^3^) are the mass of volume of oven-dried specimens, respectively.

### 2.6. Dimensional Stability

Wood dimensional stability is evaluated by swelling ratio (*S*_t,r_) in tangential or radial directions of treated and controlled wood after equalization in the conditioning chamber. The swelling ratio (*S*_t,r_) is calculated according to Equation (4).

*S*_t,r_ = 100 × (*L*_e_ − *L*_o_)/*L*_o_ (4)

where *L*_e_ is the dimensions in tangential or radial directions after equalization in the conditioning chamber, *L*_o_ is the oven-dried dimension in tangential or radial directions.

### 2.7. Bending Strength and Modulus of Elasticity

The bending strength, also termed as Modulus of rupture (MOR), and modulus of elasticity (MOE) of specimens were measured according to GB/T 1936.1-2009. The large samples of 20 (R) × 20 (T) × 300 (L) mm^3^ were first conditioned at 20 ± 2 °C and 65 ± 3% RH in a conditioning chamber until weight constancy was obtained. Thereafter, seven replicates in each group were subjected to a bending test machine (Shimadzu, Kyoto, Japan). 

### 2.8. Colour Measurements

Wood color was measured at the surface of the large samples of 20 (R) × 20 (T) × 300 (L) mm^3^ before and after HT at the same locations. Surfaces of the treated specimens were sanded down 1 mm and brushed cleanly before color measuring. The color measurements were performed using a portable sphere spectrophotometer (X-rite SP60) (Grand Rapids, MI, USA). The CIE L*, a* b* space coordinates were determined and the color changes, ΔE*, as a function of HT were calculated according to Equation (5).

ΔE* = [(ΔL*)^2^ + (Δa*)^2^ + (Δb*)^2^]^1/2^ (5)

where ΔL*, Δa*, and Δb* are the changes in lightness, green-red and blue-yellow chromatic coordinate prior to and after HT, respectively. 

### 2.9. FTIR Spectroscopy

The changes in the chemical structure [37] of control and HT wood were evaluated by means of Fourier-transform infrared spectroscopy (FTIR, VERTEX 80v) (Bruker, Rheinstetten, Germany) at a spectral resolution of 4 cm^−1^ with 16 scans. Wood powder samples were analyzed in diffuse reflectance in 4000–400 cm^−1^ region.

### 2.10. Statistical Analysis

A multiple comparison was first applied to an analysis of variance (ANOVA) using SPSS to assess the effect of HT on wood properties, and significant differences between mean values of control and treated samples were determined using Duncan’s multiple range tests (*p* < 0.05).

## 3. Results and Discussion

### 3.1. Mass Loss (ML)

**Figure 1:**
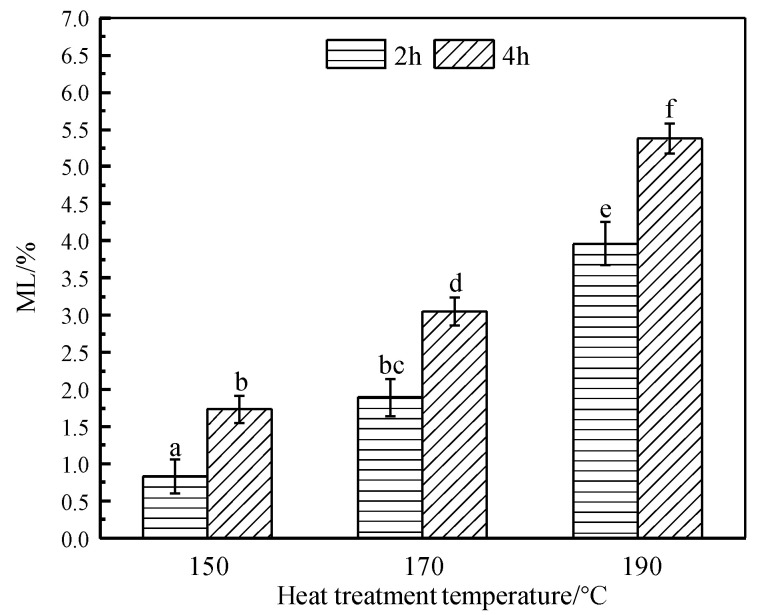
The *ML* of HT wood. Bars with different letters indicate significant differences (*p* < 0.05) according to Duncan’s multiple range tests.

The *ML* results are displayed in Figure 1, which ranged between 0.82 and 5.83% and increased with increasing temperature and duration. The effect of HT was small at 150 °C, but became significant when temperature exceeded 170 °C, especially at 190 °C. Both temperature and duration affect *ML* of wood significantly (*p* < 0.05), but the temperature is more effectively. Similar trend for *ML* was also reported in Cademartori’s study [38], which was attributed to particular thermal degradation of wood macromolecules. Among them, hemicelluloses first degrade and release acetic acid which significantly accelerates thermal degradation process of wood with increasing temperature and duration [39].

### 3.2. Moisture Adsorption

**Figure 2:**
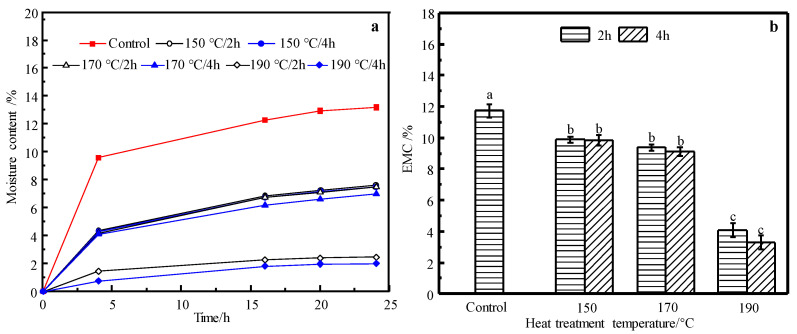
Short-term water vapor sorption process (**a**) and *EMC* after long-term water vapor sorption (**b**) of the control and HT wood. Bars with different letters indicate significant differences (*p* < 0.05) according to Duncan’s multiple range tests.

The MC changing in the short-term (24 h) and equilibrium moisture content (*EMC*) after long-term (4 weeks) vapor sorption of control and HT wood are presented in Figure 2a,b, separately, which demonstrate the capacity of wood for moisture adsorption. Figure 2a indicates the velocity of water vapor adsorption of the control and HT wood in short-term. The velocity of water vapor adsorption decreased significantly after heat treatment and varied considerably with treating severity. Meanwhile, comparing with the control wood, *EMC* decreased considerably from 11.71% in the control to 3.27% in the treatment of 190 °C/4 h, showing a decrement of 72.1%. However, for HT wood at 150 and 170 °C, there were no significant differences (*p* < 0.05) in *EMC*, indicating both temperature and duration has little effect of *EMC* in these temperatures. For the treatment at 190 °C, treating duration also has no significant effect (*p* < 0.05) on *EMC* of HT wood.

### 3.3. Density

**Figure 3:**
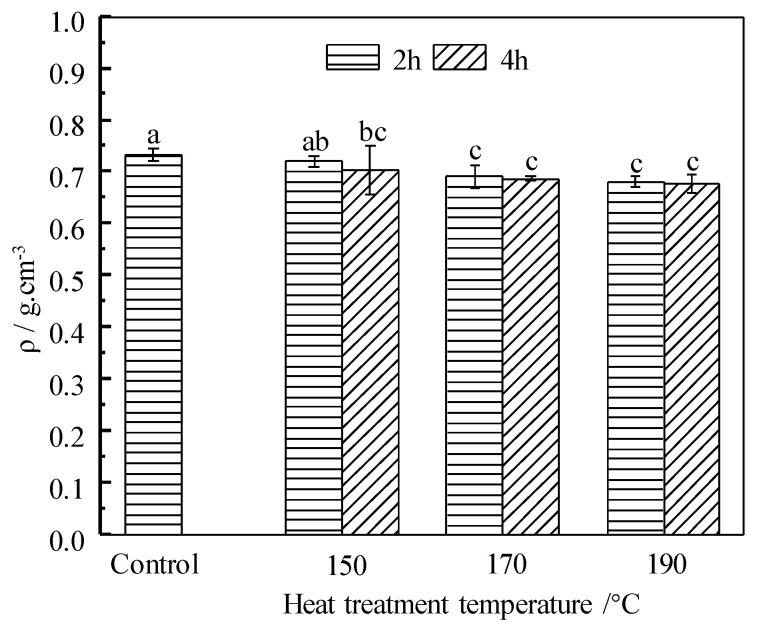
Density of the control and HT wood. Bars with different letters indicate significant differences (*p* < 0.05) according to Duncan’s multiple range tests.

Figure 3 is the absolute dry density of the control and HT wood. Comparing with the control, the absolute dry density of HT wood decreased, except the group at 150 °C/2 h. Density is defined as a ratio of mass and unit volume of wood, which is affected by both of them. *ML*s are observed after HT in Figure 1 and decreases significantly with treating severity. Thus, density reduction presents similar tendency with *ML*. Comparing the reduction of *ML* and density in the severity conditions of 170 °C/4 h and 190 °C/4 h, *ML*s are 2.01% and 5.83% respectively, but density lose are 6.14% and 7.64%, respectively. These results indicate that density loss is greater than *ML*. Although density decease after HT, there are no significant differences (*p* < 0.05) in density among the groups of 150 °C/4 h, 170 °C and 190 °C.

### 3.4. Dimensional Stability

**Figure 4:**
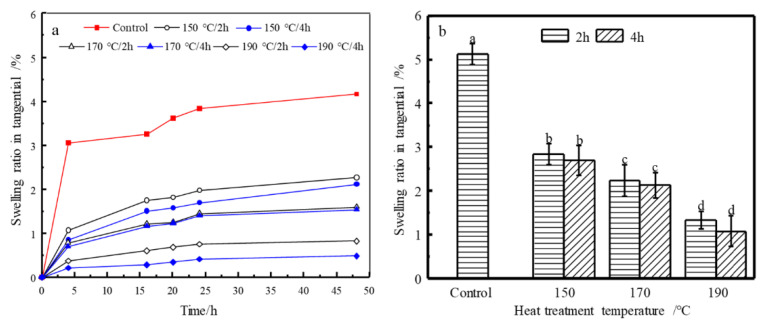
Tangential welling ratio in short-term water vapor sorption process (**a**) and at *EMC* of the control and HT wood (**b**). Bars with different letters indicate significant differences (*p* < 0.05) according to Duncan’s multiple range tests.

**Figure 5:**
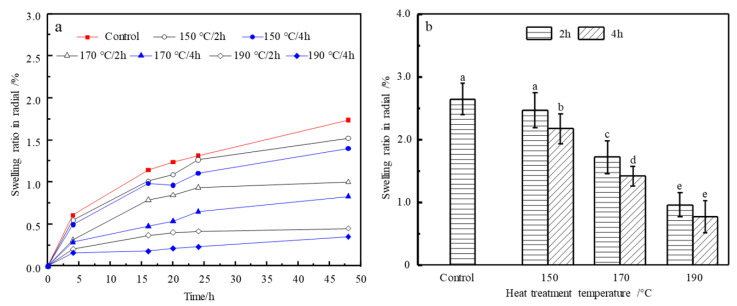
Radial swelling in short-term water vapor sorption process (**a**) and at *EMC* of the control and HT wood (**b**). Bars with different letters indicate significant differences (*p* < 0.05) according to Duncan’s multiple range tests.

The tangential swelling ratio in the short-term (48 h) and at *EMC* after long-term (4 weeks) vapor sorption of control and HT wood are shown in Figure 4a,b, respectively, which demonstrate the dimensional stability of control and HT wood. Figure 5a,b is the radial swelling ratio. In the short-term process, all groups swelled fast in the first 4 h and then became slowly. There were very few swellings from 24 h to 48 h. The swelling behavior of HT wood was different from the control wood. The swelling of the control wood is slow and decreases significantly with HT severity. After all samples reached *EMC*, there are significant reductions in the swelling of HT wood. Compared to control wood, the tangential swelling ratios of HT wood at 150, 170, and 190 °C decreased by 46.15%, 57.52%, and 76.56%, respectively. Thus, heat treatment improves dimensional stability of wood remarkably. However, in same treating temperatures, treating duration has no significant effect (*p* < 0.05) on tangential swelling (Figure 4b).

The radial swelling of HT wood present similar trend with that in tangential direction, i.e., swellings decrease after HT and become significant with the severity of treating temperature and duration. Comparing with control wood, the radial swelling ratios of HT wood at 150, 170, and 190 °C decreased by 12.56%, 40.70%, and 67.24%, respectively. The reduction is slightly smaller than that in tangential direction, except the 150 °C conditions, which resulted in small reduction of swelling.

**Figure 6:**
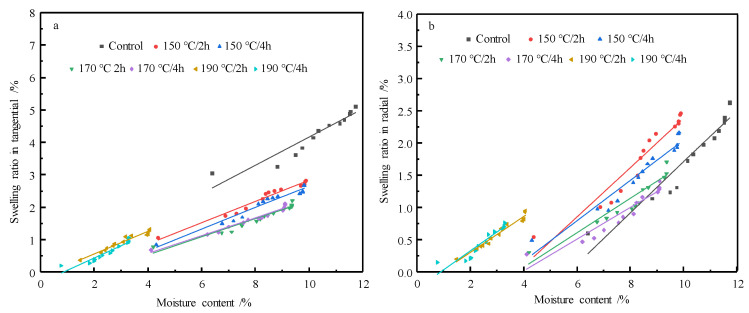
Relation between welling ratio and moisture content of control and HT wood in tangential (**a**) and radial (**b**) direction.

The relation between swelling ratio and moisture content of control and HT wood are shown in Figure 6a,b. The swelling ratio and moisture content present a linear relationship for both the control and HT wood, indicating HT does not affect fundamental characteristics of wood shrinkage. The regression equations of swelling versus moisture content are summarized in Table 1. The coefficients of the regression equations are the swelling coefficient of wood, which demonstrate the swelling properties wood material. The smaller swelling coefficient, the less welling of wood. The swelling coefficients of HT wood in tangential and radial direction decrease in contrast to the control wood. This also indicates the swelling of HT wood are smaller than the control and wood dimensional stability improves after HT. However, the effects of treating conditions are different, and the swelling coefficient of wood treated at 170 °C was the smallest.

### 3.5. Modulus of Rupture and Modulus of Elasticity

**Figure 7:**
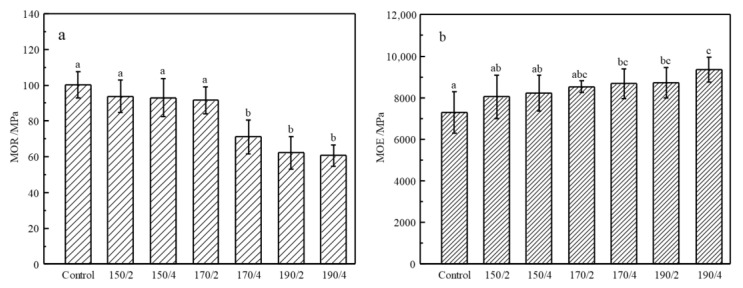
MOR (**a**) and MOE (**b**) of the control and HT wood. Bars with different letters indicate significant differences (*p* < 0.05) according to Duncan’s multiple range tests.

Figure 7 is the MOR and MOE of the control and HT wood. In contrast to the control, the MOR of HT wood at 150 °C/2 h, 150 °C/4 h, and 170 °C/2 h have no significant decrease (*p* < 0.05), however, they decrease significantly (*p* < 0.05) when treating conditions became severe. For the 190 °C conditions, MOR decreased by 31.7% compared with the control wood. The reduction of MOR in severe HT is in agreement with the previous report [40]. The reduction of MOR is mainly attributed to the degradation of hemicellulose [41,42]. In contrast, the bending stiffness, MOE, presented an obviously opposite trend. There were no significant (*p* < 0.05) improvements of MOE between control and HT wood at 150 °C/2 h, 150 °C/4 h, and 170 °C/2 h. However, MOE of HT wood t at 170 °C/4 h, 190 °C/2 h, and 190 °C/4 h increased significantly (*p* < 0.05) compared with the control wood. For the 190 °C conditions, there was a 19.64% improvement of MOR compared with the control wood. Despite of the changes of MOR or MOE, wood generally become brittle after heat treatment which is a problem for its further processing.

### 3.6. Colour Change

The changes of color coordinate in a*, b*, L*, and ΔE* of wood before and after HT are presented in Table 2. Δa* decreased with HT severity and presented minus values, indicating wood color become green after HT. In contrast, Δb* showed an opposite tendency, which increased with HT severity and presented plus values, this means wood color become more yellow along with treating severity. Lightness changes affect wood color remarkably. ΔL* presenting minus values decreased significantly with treating severity, indicating that wood color become darker along with the severer treating. The darkening of wood is attributed to the increase of lignin content in the cell walls, but also to the depolymerization of the hemicelluloses and the increase of low molecular weight sugars produced there from [43]. The total color changes ΔE* of wood are small at 150 °C, become greater with the improvement of temperature, and reached the maximum value at 190 °C/4 h. For the 150 and 170 °C conditions, effects of treating duration on ΔE* are not obvious, however it became significant at the severer conditions in 190 °C. Color of eucalyptus urophylla became dark, but the color deviation also became small, indicating wood color is more uniform. The darken color benefits for its further application in indoor decoration products, but the color of HT wood is not UV-stable.

### 3.7. Changes in the Chemical Structure

**Figure 8:**
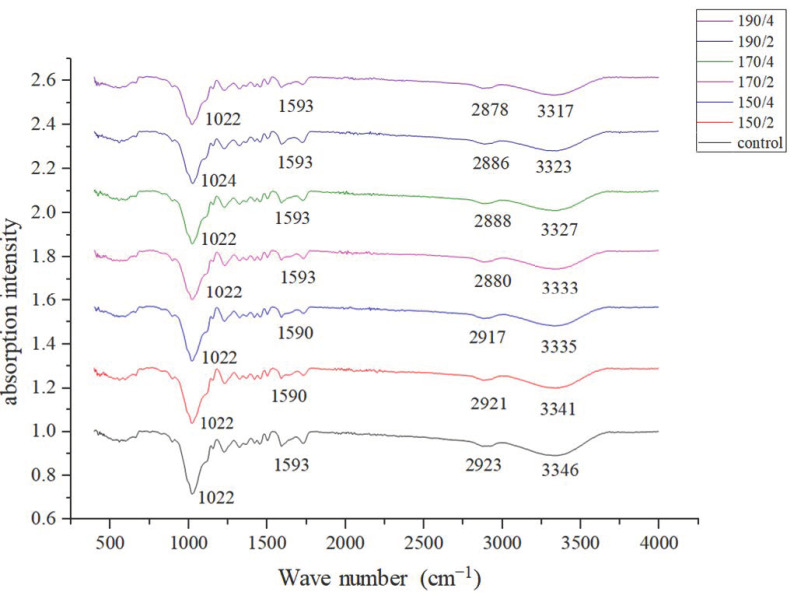
Infrared spectra of the control and heat-treated wood.

The Figure 8 shows the infrared spectrum image of the control and HT wood. The curves in the figure have similar absorption intensity peaks. The wave members are around 3400 cm^−1^, 2900 cm^−1^, 1600 cm^−1^, and 1050 cm^−1^, which demonstrate the stretching vibration peak of –OH, C–H bond, C=O bond as well as benzene ring of lignin, and C–O bond, respectively. Additionally, the peak at 1270 cm^−1^ is the stretching vibration peak of the structural unit of guaiacyl and the peak at 1505 cm^−1^ is corresponding to stretching vibration of the lignin benzene ring [44]. Compared with the control wood, the intensity of –OH group at 3400 cm^−1^ of HT wood deceased, indicating heat treatment reduce the relative content of hydroxyl groups of wood components. The reduction of hydroxyl groups decreases water absorption capacity of wood, thereby improving dimensional stability of wood. There are almost no changes in the absorption peaks of C–O, C=O, and C–H, indicating that heat treatments have little effect on them. Color changes of wood are related to the chromophoric groups, such as carbonyl, carboxyl, and alkene conjugated with benzene ring of lignin. There were no significant changes in the peak at 1505 cm^−1^, indicating lignin benzene ring remained basically stable after heat treatment. However, the changes at 1600 cm^−1^ absorption peak occur, which are attributed to the conjugated carbonyl group connected to the benzene ring due to the heat treatment [45]. In addition, the stretching vibration peak of the structural unit of guaiacyl near 1270 cm^−1^ also has a slight decrease in peak intensity. All these changes indicate the chromophoric groups and its own structure of lignin varied due to the heat treatment, which affects color of wood.

## 4. Conclusions

*Eucalyptus urophylla* S.T. Blake wood were heat treated at 150, 170, and 190 °C, for 2 and 4 h, respectively. The physical and mechanical properties, wood color, dimensional stability, and chemical change of HT wood were investigated. The results are summarized as: Mass loss (*ML*) of wood at a moderate temperature of 150 °C was small, but increased remarkably when temperature exceeds 170 °C and the maximum reduction of *ML* is 5.83% for the conditions of 190 °C/4 h; both velocity of water vapor adsorption and equilibrium moisture content (*EMC*) of HT wood decreased significantly, and they varied considerably with treating severity. *EMC* of HT wood at 190 °C/4 h is 3.27%, which decreased by 72.10% in contrast to the control wood; absolute dry density of HT wood decreased, showing a similar tendency with *ML*, but the reduction was greater than *ML*; The tangential and radial swelling ratio and swelling coefficients of HT wood declined, and the dimensional stability of HT wood at 190 °C conditions was improved by 71.88%; there were no obvious changes in MOR and MOE of HT wood in a moderate condition. MOR of HT wood decreased significantly at the severer conditions, but MOE of HT wood increased performing inversely with MOR. Despite of the changes of MOR or MOE, wood generally becomes brittle after heat treatment; wood color changed slightly in the 150 °C conditions, but it became darker and uniform after severe treating. Wood’s color after HT is not UV-stable; the relative content of hydroxyl groups in wood components was reduced after HT, resulting in improvement of wood dimensional stability. Color change of wood may be caused by variations of chromophoric groups and its own structure of lignin, as well as depolymerization of the hemicelluloses owing to heat treatment. Moderate temperature HT at 150 °C improves wood dimensional stability and color uniform but without negatively reducing mechanical stress. This is a practical HT condition for *Eucalyptus urophylla* S.T. Blake.

## Figures and Tables

**Table 1:** Regression Equations and Correlation Coefficient of Swelling Ratio and Moisture Content of Control and HT Wood.

Treating Conditions		Tangential		Radial	
Temperature (°C)	Time (h)	Regression Equations	Correlation Coefficient	Regression Equations	Correlation Coefficient
-	-	Y = 0.4354X − 0.1759	0.8949	Y = 0.3963X − 2.2416	0.9169
150	2	Y = 0.3249X − 0.407	0.9743	Y = 0.3846X − 1.445	0.9226
	4	Y = 0.3312X − 0.6439	0.9621	Y = 0.3139X − 1.0778	0.9394
170	2	Y = 0.28X − 0.5751	0.9327	Y = 0.2664X − 0.9818	0.9012
	4	Y = 0.2763X − 0.5193	0.9742	Y = 0.2454X − 0.9569	0.9018
190	2	Y = 0.3426X − 0.1015	0.9550	Y = 0.2792X − 0.2493	0.9594
	4	Y = 0.3778X − 0.2898	0.8984	Y = 0.2918X − 0.2489	0.8777

**Table 2:** Effect of Heat Treatment on Wood Color.

Treating Conditions		Δa*	Δb*	ΔL*	ΔE*
Temperature/°C	Time/h				
150	2	−1.17	1.45	−3.98	4.4
	4	−0.66	0.68	−4.89	5.0
170	2	−2.30	2.17	−8.39	9.0
	4	−1.34	1.85	−10.59	10.8
190	2	−3.20	6.71	−16.67	18.3
	4	−3.08	5.27	−22.53	23.3

## Data Availability

Not applicable.
